# Drying characteristics of yam slices (*Dioscorea rotundata*) in a convective hot air dryer: application of ANFIS in the prediction of drying kinetics

**DOI:** 10.1016/j.heliyon.2020.e03555

**Published:** 2020-03-11

**Authors:** John O. Ojediran, Clinton E. Okonkwo, Abiola J. Adeyi, Oladayo Adeyi, Abiola F. Olaniran, Nana E. George, Adeniyi T. Olayanju

**Affiliations:** a Department of Agricultural and Biosystems Engineering, College of Engineering, Landmark University, P.M.B 1001, Omu-Aran, Nigeria; b Department of Mechanical Engineering, Ladoke Akintola University of Technology, Ogbomoso, Oyo State, Nigeria; c Department of Chemical Engineering, College of Engineering, Landmark University, P.M.B 1001, Omu-Aran, Nigeria; d Department of Food Science, College of Agricultural Science, Landmark University, P.M.B 1001, Omu-Aran, Nigeria

**Keywords:** Food science, Food technology, Computer science, Adaptive neuro fuzzy inference system (ANFIS), Drying, Moisture ratio, Effective diffusivity, Activation energy, Rehydration ratio

## Abstract

This study applied Adaptive Neuro-Fuzzy Inference System (ANFIS) to predict the moisture ratio (MR) during the drying process of yam slices (*Dioscorea rotundata*) in a hot air convective dryer. Also the effective diffusivity, activation energy, and rehydration ratio were calculated. The experiments were carried out at three (3) drying air temperatures (50, 60, and 70 °C), air velocities (0.5, 1, and 1.5 m/s), and slice thickness (3, 6, and 9 mm), and the obtained experimental data were used to check the usefulness of ANFIS in the yam drying process. The result showed efficient applicability of ANFIS in predicting the MR at any time of the drying process with a correlation value (R^2^) of 0.98226 and root mean square error value (RMSE) of 0.01702 for the testing stage. The effective diffusivity increased with an increase in air velocity, air temperature, and thickness and the values (6.382E -09 to 1.641E -07 m^2^/s). The activation energy increased with an increase in air velocity, but fluctuate within the air temperatures and thickness used (10.59–54.93 KJ/mol). Rehydration ratio was highest at air velocity×air temperature×thickness (1.5 m/s×70 °C × 3 mm), and lowest at air velocity × air temperature×thickness (0.5 m/s×70 °C × 3 mm). The result showed that the drying kinetics of *Dioscorea rotundata* existed in the falling rate period. The drying time decreased with increased temperature, air velocity, and decreased slice thickness. These established results are applicable in process and equipment design, analysis and prediction of hot air convective drying of yam (*Dioscorea rotundata*) slices.

## 1 Introduction

Yam (*Dioscorea spp*.) has been identified as one of the most important food crops for a wide range of tropical countries including Nigeria, Ghana, Togo, Burkina Faso, Cote d’Ivoire, with over 600 species, in which only a few are cultivated for food purpose (Olatoye and Arueya, 2019). It has been described as a staple food for 60–100 million people in the world, also serves as a good source of carbohydrate (Amandikwa et al., 2015). Due to the high moisture content (50–80 % wet basis) of yam and its susceptibility to deterioration during storage, it is difficult to store fresh yams (Chen et al., 2017; Falade and Onyeoziri, 2012). Drying has been regarded by humans as probably the most important and oldest food preservation method and it entails a complex thermal process in which simultaneous heat and mass transfer occur (Ojediran and Raji, 2010; Doymaz, 2011). It is a process of moisture reduction in agricultural products to extend its shelf life (Abbaspour-Gilandeh et al., 2019). High moisture content in food products increases the activities of micro-organisms, chemical, and biochemical reactions (Kaveh et al., 2018a). During drying of a wet agricultural product, two phenomena occur simultaneously; transfer of heat energy to the product and movement of internal moisture to the surface of the product were it is evaporated. Dried white yam can be stored for a longer period, used as instant yam flour for cooking, and extraction of resistant starch (Falade and Onyeoziri, 2012; Srikanth et al., 2019). The three (3) major drying processes based on heat transfer are; conduction, convection, and radiation (Liu et al., 2019a). Some of the drying technologies which have been used for agricultural products include; sun drying, hot air convective drying, vacuum drying, microwave drying, infrared drying and their mixtures (Omari et al., 2018; Kaveh et al., 2018a). Hot air drying which has two major importance; efficient removal surface water and low operating cost, involves blowing heated air over food materials to remove moisture has been used frequently in food dehydration (Jimoh et al., 2009; Omari et al., 2018). Mathematical models have been used in describing the drying process of several food products, but it is still associated with a range of difficulties including; estimation of many experimental parameters, application of advance calculation methods, and deep knowledge of the process mechanism which the black-box modeling approach is to solve (Omari et al., 2018). In recent times, the applicability of the black-box modeling method also called soft computing technique is becoming popular partly because of their high accuracies and ease of use. They are the best fit for the situation where exact mathematical models or information is difficult to establish for the dynamics of a system. Soft computing methods include; Adaptive Neuro-Fuzzy Inference System (ANFIS), Artificial Neural Networks (ANN), Fuzzy Inference System (FIS) and Genetic Algorithms (GA) amongst others (Omari et al., 2018). The soft computing methods differ from conventional or traditional computing methods but are complementary in the sense that they are tolerant of imprecision, partial truth, approximation, uncertainty, and heuristic approach (Zalnezhad et al., 2013). Soft computing is artificial intelligent techniques, which simply refers to machine intelligence used in controlling or performing seemingly complicated tasks. Machine intelligence is important in modern-day because human brains are not able to efficiently managed exponentially growing information, hence the need for machine assistance. Application of artificial intelligence methods in drying is still in developmental stages and its continued development is anticipated in other to cater to new needs and new solutions; therefore, studies in this area are still relevant. The gap inapplicability of artificial intelligent techniques has been partly attributed by Martynenko (2017) to the lack of commercially available artificial intelligent systems for drying of agricultural products and bio-related materials. This is a result of difficulties associated with the interpretation of artificial intelligence language for the realistic needs of drying community or probably due to the availability of simpler but less accurate alternatives such as Proportional Integral Derivative controller.

In some previous studies concerning the applicability of artificial intelligent methods; Zalnezhad et al. (2013) recorded a good approximation in application of fuzzy logic for the prediction of surface hardness of alloy coating; Yousefi (2017) applied ANFIS and genetic algorithm artificial neural network (GA-ANN) to modeling the drying kinetics (MR) of papaw slices in a hot air dryer. Results showed that ANFIS has a better prediction ability, based on the implications of statistical root mean square error (RMSE) values; Kumar and Sharma (2016) used response surface methodology (RSM) and ANFIS in modeling the extraction process of bioactive compounds from taro, the results obtained using ANFIS was found to be competitive to RSM; Rahman et al. (2012), compared the use of ANFIS, multivariable regression, and ANN in prediction of the thermal conductivity of foods, ANFIS was reported to provide a better prediction ability as compared to ANN and multivariable regression; Kaveh et al. (2018b) applied ANN and ANFIS for prediction of the drying characteristics (moisture diffusivity, moisture ratio, drying rate) and specific energy consumption (SEC) of potato, garlic, and cantaloupe in a hot air convective dryer, ANFIS was showed to have higher prediction ability than ANN; Al-Mahasneh et al. (2016), conducted a review on the application of artificial intelligent modeling tool like; ANFIS, ANN, fuzzy inference system (FIS), and multiple linear regression (MLR) in food processing and technology, ANFIS was reported to produce a better performance. Pusat et al. (2015), used ANFIS in the prediction of moisture content of coal in a convective drying process; the predicted result was reported to be in an agreement with the experimental data with a high correlation coefficient. Abbaspour-Gilandeh et al. (2019), used ANN and ANFIS in the prediction of the kinetic, energy and exergy of quince fruit in a hot air dryer. The prediction ability of ANFIS was compared with ANN and other mathematical models, ANFIS had the best performance. Kaveh et al. (2018b) compared the use of ANFIS, ANN, and other eleven (11) mathematical models in prediction of moisture ratio of an almond kernel in a convective dryer, ANFIS had the best prediction ability. Liu et al. (2019a) applied ANN in the prediction of energy and exergy of mushroom during drying in a hot air impingement dryer. Liu et al. (2019b) used extreme learning machine (ELM) in the prediction of the drying behavior of broccoli florets. Liu et al. (2019c) used Bayesian extreme learning machine (BELM) in color prediction of mushroom during drying.

In this study, ANFIS was used to simulate the moisture ratio drying characteristics of yam (*Dioscorea rotundata*) slices in a hot air convective dryer. Moisture ratio was specifically selected because it forms the background on which other drying characteristics including drying rate, effective diffusivity, and activation energy amongst others are based. Hence, understanding the dynamics of moisture ratio will improve the accurate representation and analysis of the other drying characteristics. The choice of ANFIS was made because of its hybridized nature leading to high accuracy tendencies more than the un-hybridized methods. Also, ANFIS or any other soft computing tool has not been reportedly used in modeling the drying kinetics of yam (*Dioscorea rotundata*) from literature, and this formed the basis of this study. The aim or objective of this study was to (1) use ANFIS in modeling the moisture ratio (MR) of yam slices in a convective hot air tray dryer (2) study the effect of temperature, air velocity, and thickness on the effective diffusivity, activation energy, and rehydration ratio of yam.

## 2 Materials and method

### 2.1 Materials

White yam (*Dioscorea rotundata*) used for this study was purchased from a local market and identified at the Landmark University Teaching and Research Farm, Omu-Aran in January 2019. Tubers of size (0.5–1 kg) free of injuries were selected. The initial moisture content of the yam was determined using the AOAC (1980) method, and the value was obtained to be 66.70 ± 0.7 % (wet basis). The yam was washed and peeled manually with a stainless knife into rectangular sizes of varying thickness as presented in Table 1.

**Table 1:** Experiment conditions.

S/N	Air temperatures (^°^C)	Air velocity (m/s)	Slice thickness (mm)
1	50	0.5	3
2	60	1	6
3	70	1.5	9

### 2.2 Method

**Figure 1:**
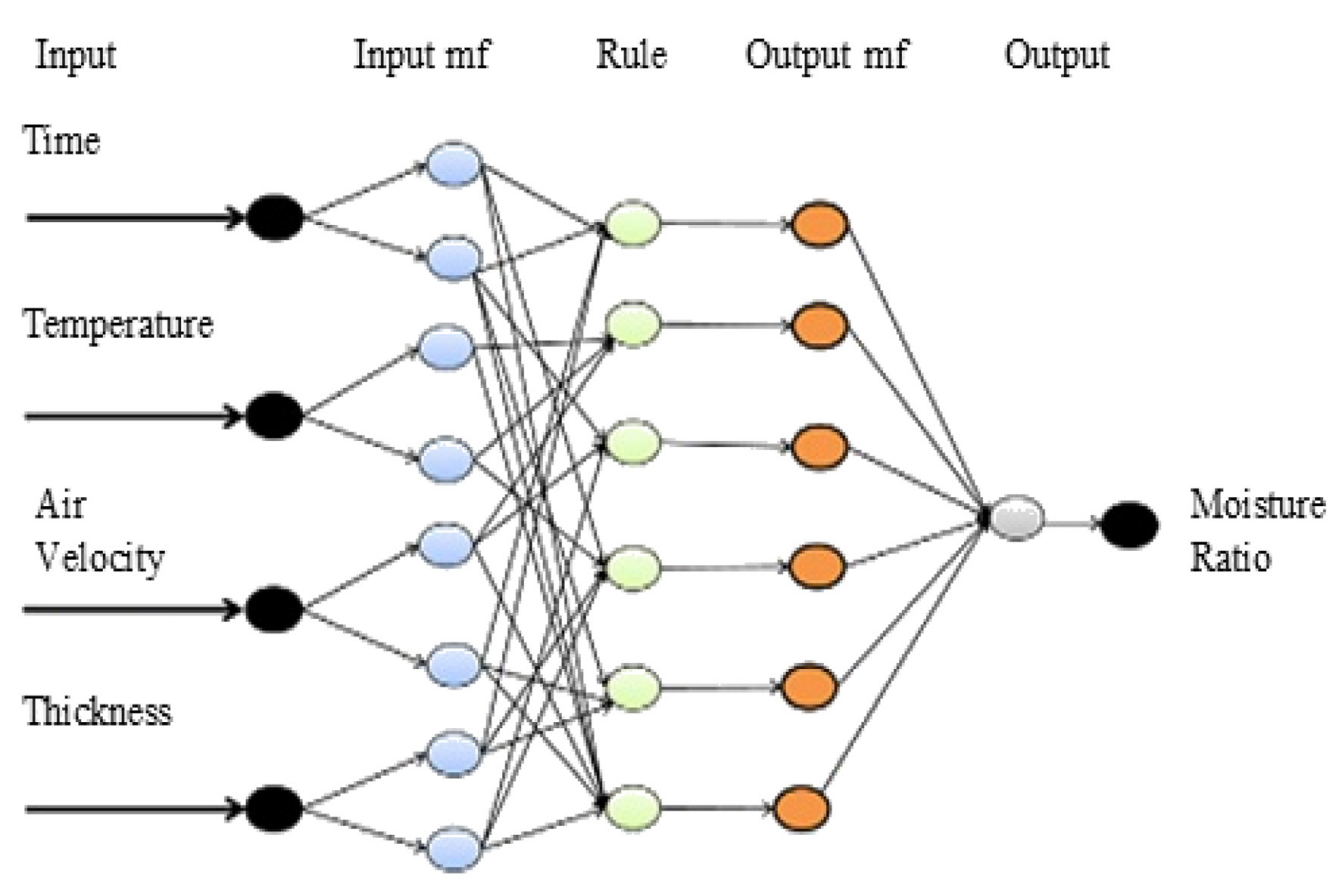
ANFIS structure in this study.

The summary of the experimental data used in this study is represented in Table 2 and the developed ANFIS structure from the experimental data is shown in Figure 1. There are 4 inputs (drying time, air temperature, air velocity, and yam slice thickness) and 1 output (moisture ratio).

**Table 2:** Summary of experimental drying data.

Statistical Term	Time (min)	Temperature (^°^c)	Thickness (mm)	Air velocity (m/s)	Moisture Ratio (%)
Minimum	0.0000	50.0000	3.0000	0.0000	
Maximum	780.0000	70.0000	9.0000	1.5000	1.1489

#### 2.2.1 Drying procedure

**Figure 2:**
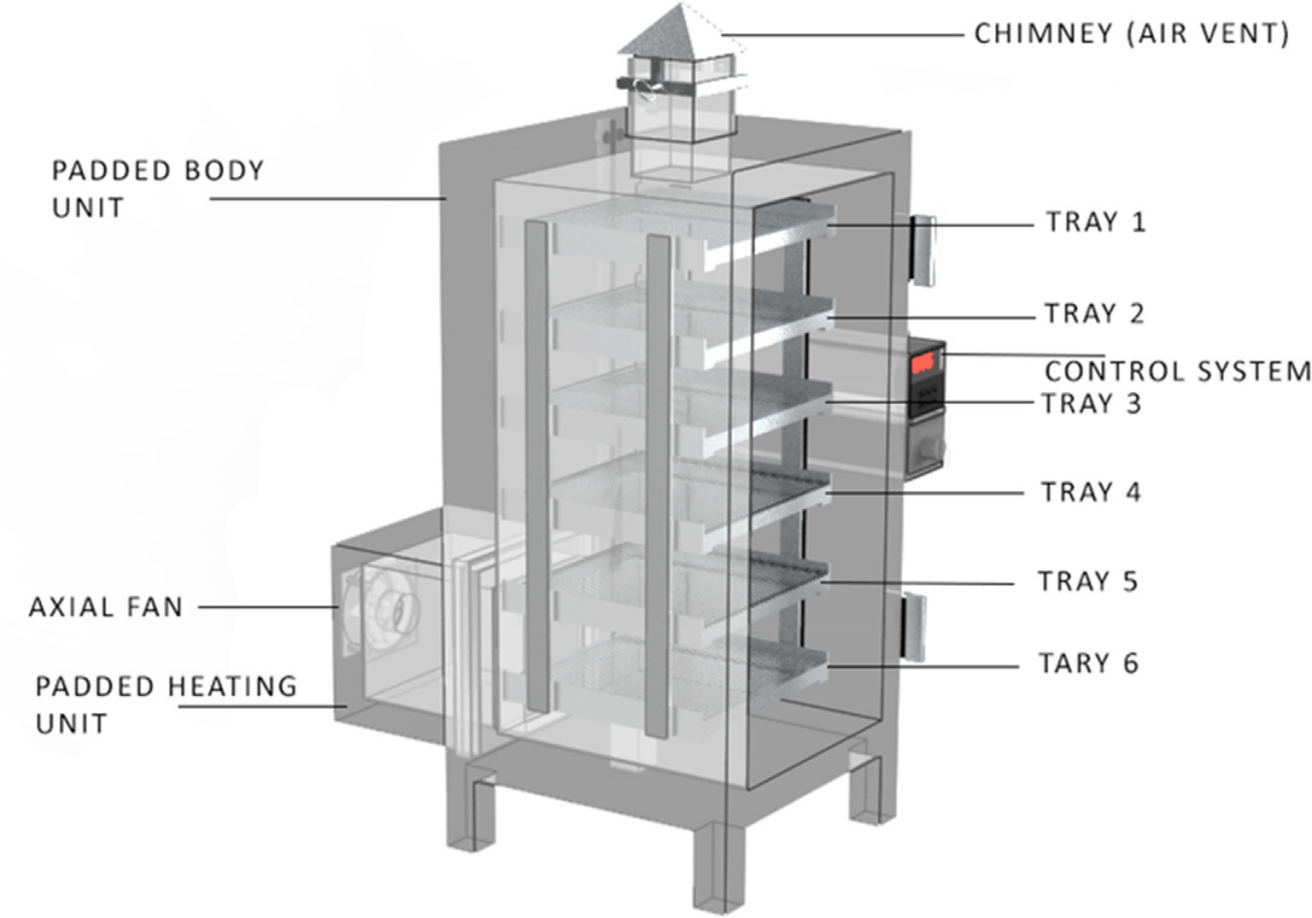
Schematic diagram of the fabricated drier used for the drying of the yam slice.

Yam samples were introduced into the hot air drier (Figure 2) developed at the Department of Agricultural and Biosystems Engineering, Landmark University, Omu-Aran, Nigeria. The dryer was allowed to run for about 30 min on zero loads to reach the desired experimental conditions presented in Table 1 before the drying experiment commenced. Samples were weighed before being introduced into the drier and removed at interval of 10 min for the first 1 h, 30 min for the next 2 h, 1 h for the next3 hours, and 2 h for subsequent drying times until three (3) constant consecutive weights were noticed indicating equilibrium condition (Falade et al., 2007; Ojediran and Raji, 2010). The drying experiment was carried out in five replicates and the mean values obtained.

#### 2.2.2 Description of hot air dryer

Some of the essential parts of the dryer include; an adjustable axial fan, air heating chamber (1 kW), drying chamber, control system, chimney (moist air exit), and trays. The dryer has a control system to set the desired temperature and control the air velocity. The axial fan was directed parallel to the heating source, and the hot air produced in this chamber was blown into the drying chamber where the products are contained. The moist air produced from the yam slices during drying leaves the dryer through the chimney located at the top of the dryer. The drying temperature was measured using a thermocouple (M6 Screw thermocouple KE PT100 type, temperature sensor, China) having an accuracy of ±0.3^o^c and it is inserted at the center of the dryer which triggers off the temperature controller (STEL XMTD-2001, accuracy of 1.0, range; 0-399^o^c) once the internal temperature has reached the set temperature. Air velocity used was between 0.5 – 1.5 m/s. The hot air blows perpendicular to the direction of the sample. The dimension of the drying chamber is0.4×0.8m, having six trays with a capacity of 0.5 kg. Dried samples were manually weighed using an analytical semi-micro balance having accuracy ±0.0001g, readability of 0.1mg, and maximum capacity, 210 g (AND GR-200, Japan).

#### 2.2.3 Drying kinetics

Drying kinetic reveal good information about the drying process of yam slices (Omari et al., 2018).

Moisture ratio (MR) for yam slice was calculated using Eq. (1) (Omari et al., 2018).

(1) $$MR=\frac{M_{t}-M_{e}}{M_{o}-M_{e}}$$

Where$\;M_{t}$, is the moisture content at any time of drying (kg water/kg dry matter), $M_{e}$ is the equilibrium moisture content (kg water/kg dry matter), $M_{o}$ is the initial moisture content (kg water/kg dry matter).

The drying rate (DR) for yam slice was calculated using Eq. (2) (Ju et al., 2016).

(2) $$DR=\frac{M_{t+dt}-M_{t}}{dt}$$

Where $M_{t+dt}$ is the moisture content at t+dt (kg water/kg dry matter), t is time (min), dt is the time difference (min).

#### 2.2.4 ANFIS modeling

Practical systems are complex and for a complex system to function optimally, model or it structural or mathematical representation becomes necessary, this explains the increased modeling practice in modern science (Mrinal, 2008). Modeling systems with the use of conventional mathematical tools are not well acceptable for dealing with indistinct and undetermined systems (Jang, 1993); however, intelligent modeling methods show great ability in this regard. An amalgamated or hybrid intelligent system like ANFIS (i.e. fuzzy inference system - artificial neural network) is a subset of other individual intelligent systems. ANFIS functionality combines the openness of a fuzzy inference system with the learning ability of artificial neural networks. In its theory, ANFIS has a structure comprising of a back-propagation algorithm that is bound with multi-layer neural network cum Sugeno fuzzy type with input and output layers with three hidden layers (Dideková and Kajan, 2009). The hidden layers are for input membership function, rules, and output membership function respectively. The adaptive network usually consists of nodes and directional links, which are the connectors of the node. The nodes are truly adaptive because they depend on the node parameters, and during learning; these parameters are changed based on the learning rules with a focus on minimizing a prescribed error measure. The specific ANFIS structure concerning this study is as represented in Figure 2.

It consists of four inputs-one output system. The single output is typical of the Sugeno fuzzy inference system. The fuzzy rules are generally established by logically linking the input and output parameters. A common rule set for this structure can be formulated as (Kaveh et al., 2018b; Abbaspour-Gilandeh et al., 2019):

Rule 1: if T is AA_1_, V is BB_1_, DT is CC_1_ and PT is DD_1_ then

(3) $$f_{1}=p_{1}\text{T}+q_{1}\text{V}+r_{1}\text{DT}+s_{1}\text{PT}+u_{1}$$

Rule 2: if T is AA_2_, V is BB_2_, DT is CC_2_ and PT is DD_2_ then

(4) $$f_{2}=p_{2}\text{T}+q_{2}\text{V}+r_{2}\text{DT}+s_{2}\text{PT}+u_{2}$$

where T is the drying time (min), V is the temperature (°C), DT is the air velocity (m/s) and PT is the yam slice thickness (mm), *f* is the moisture ratio, AA_1_, AA_2_, BB_1_, BB_2_, CC_1_, CC_2_, DD_1_, and DD_2_ are the language indicators, r1, r2, q1, q2, p1, p2, s1, s2, u1, and u2 are the linear coefficients of the output function of which *f*_1_ and *f*_2_ are first-degree polynomials. The functions of layer 1, 2, 3, 4 and 5 are fuzzification, multiplication, normalization, defuzzification, and summation, respectively and they can be explained as follow:

Layer 1: square node equipped with node function

(5) $${O_{i}}^{1}=\left(\text{inputs}\right)$$

The inputs are transformed into fuzzy sets through the selected membership functions (triangle, generalized bell-shaped, Gaussian membership). *O*_*i*_^1^is the layer, and *μ*_Ai_ is the membership function of the linguistic label connected with the node function.

Layer 2: This node multiplies the incoming signal and sends the product out. Each node output is the firing strength of a rule.

(6) $${O_{i}}^{2}=wi=\mu_{A}\left(\text{input}\;1\right)\times \mu Ai\left(\text{input}\;2\right),\ldots \ldots i=1\text{,}2$$

Layer 3: circle node. The node computes the ratio of i-th rule's firing strength to the sum of all rules' firing strengths:

(7) $${O_{i}}^{3}=’=wi/w1+w2,\;i=1,\;2$$

*w*’ is the normalization rate and each of the *wi* values represents a small scale of *wi* in the layer.

Layer 4: Square node with node function:

(8) $${O_{i}}^{4}=w_{i}^{\prime }f_{i}=w^{\prime }\left(p_{i}x+q_{i}y+r_{i}\right),\;i=1,\;2$$

p, q, r – parameter set (consequent, linear, parameters).

Layer 5: circle node. This node computes the overall output as the summation of all incoming signals.

(9) $${O^{5}}_{i}=\text{overall}\;\text{output}=\sum iw^{’}if=\sum iwifi/\sum ,=1,\;2$$

ANFIS is a data driving modeling tool. Its modeling and prediction activity involves the usage of training and checking data sets. The training data set is used for architectural development while the testing data set is used for the determination of the effectiveness of the developed structure. It is therefore important that such data used, is a true representation of the system under consideration with minimal noise inclusion. In this study, Matlab 2014b software was used for the ANFIS analysis of the drying data. The data set consisted of four (4) input by one (1) output system. The input is the drying time (s), sample thickness (mm), air velocity (m/s) and drying temperature (^°^C) while the output is the sample moisture content (%). The summary of the experimental data used in this study is represented in Table 2. The performance of ANFIS was evaluated based on the comparison between the predicted and experimental value using a statistical evaluator consisting of Root Mean Square Error (RMSE) and Coefficient of determination ($R^{2})$ given Eqs. (10) and (11) (Khodabakhsh et al., 2015). The statistical parameters are calculated based on the following mathematical representation stated below:

(10) $$\text{Coefficient}\;\text{of}\;\text{determination}\;\left(R^{2}\right)=1-\sum_{i=1}^{N}\frac{{\left(\text{Pred},\text{i }–\text{ Exp},\text{ i}\right)}^{2}}{{\left(\text{Pred},\text{i }–\text{ AverageExp}\right)}^{2}}$$

(11) $$\text{Root}\;\text{Mean}\;\text{Square}\;\text{Error}\;\left(RMSE\right)=\sqrt{\frac{\sum_{i=1}^{n}{\left(\text{Exp},\text{i }–\text{ Pred},\text{ i}\right)}^{2}}{N}}$$

Where Pred, i is the ith predicted value, Exp, i is the ith experimental value and AveragedExp is the average of all the experimental value. N represents the number of observations.

#### 2.2.5 Effective diffusivity

In food products, water moves through the pores to the surface via diffusion of liquid water under the effect of concentration gradient during drying (Koua et al., 2009). The effective diffusivity reveals the mechanism of water transport through products (Ju et al., 2016). The moisture diffusion coefficient in addition to the one-dimensional Fick's diffusion law generally used was theoretically calculated from Fick's second law, and it describes the falling rate diffusion process during drying as in Eq. (12) (Ju et al., 2016; Chakraborty et al., 2016).

(12) $$\frac{\partial \left(MC\right)}{\partial t}=D_{eff}\frac{\partial {\left(MC\right)}^{2}}{\partial r^{2}}$$

The initial and boundary conditions may be detailed as follows;

At t = 0, $=MC_{i}$ , for 0<a<2b.

At t > 0, $MC=MC_{e}$, for a = 2b (top, evaporating surface).

At t > 0, $\frac{\partial \left(MC\right)}{\partial a}=0$, for a = 0 (bottom, non-evaporating surface).

For slab geometry, the general series of Fick's second law solution has been used, with some assumptions which include; negligible external resistance, negligible shrinkage, uniform moisture distribution, and constant diffusivity. Eq. (13) represents the series solution of Fick's second law for yam slice (Doymaz, 2011; Bezbaruah and Hazarika, 2014).

(13) $$MR=\frac{8}{\pi^{2}}\sum_{n=0}^{\infty }\frac{1}{{\left(2n+1\right)}^{1}}\text{exp}\left(-\frac{{\left(2\text{n}+1\right)}^{2}{\text{π}}^{2}{\text{D}}_{\text{eff}}\text{t}}{4{\text{L}}^{2}}\right)$$

Where n is taken as 1 for longer drying times (Doymaz, 2011), ${\text{D}}_{\text{eff}}$ is the effective moisture diffusivity (m^2^/s), t is the drying time (s), L is considered as half-thickness of yam slice (m).

Eq. (14) is the logarithmic form of Eq. (13), used for long drying times

(14) $$lnMR=\ln\left(\frac{8}{\pi^{2}}\right)-\left(\frac{\pi^{2}D_{eff}t}{4L^{2}}\right)$$

When lnMR is plotted against drying time (t), it produces a slope (K) which can be used to determine the effective diffusivity in Eq. (15) (Doymaz, 2011).

(15) $$K=\frac{\pi^{2}D_{eff}}{4L^{2}}$$

#### 2.2.6 Activation energy

The Arrhenius equation which describes the dependence of the effective moisture diffusivity to the temperature was used in calculating the activation energy of white yam slice as shown in Eq. (16) (Doymaz, 2011; Srikanth et al., 2019).

(16) $$D_{eff}=D_{0}exp\left(-\frac{E_{a}}{RT_{a}}\right)$$

Where $D_{0}$ is the Arrhenius equation pre-exponential factor, (m^2^/s), $E_{a}$ is the activation energy (KJ/mol), R is the universal gas constant which is usually 8.3143 KJ/molK, $T_{a}$ is the absolute temperature (i.e. T^o^c+273.15).

#### 2.2.7 Rehydration ratio

Rehydration of food products explains the ability of dried samples to re-absorb moisture when soaked in water. Dried white yam slices were hydrated in distilled water at 50 °C for 30 min in five replicates as described in the rehydration experiment carried out by Song et al. (2018). The rehydrated slice was drained on a mesh for 60s to remove superficial water, weighed using an electric balance (AND GR-200, Japan) having a sensitivity of ±0.0001g, and the rehydration ratio (RR) was calculated with Eq. (17).

(17) $$RR=\frac{M_{f}}{M_{O}}$$

Where $M_{f}$ is the weight of the rehydrated sample (g), the weight of the dry sample (g).

## 3 Results and discussion

### 3.1 ANFIS modeling

**Figure 3:**
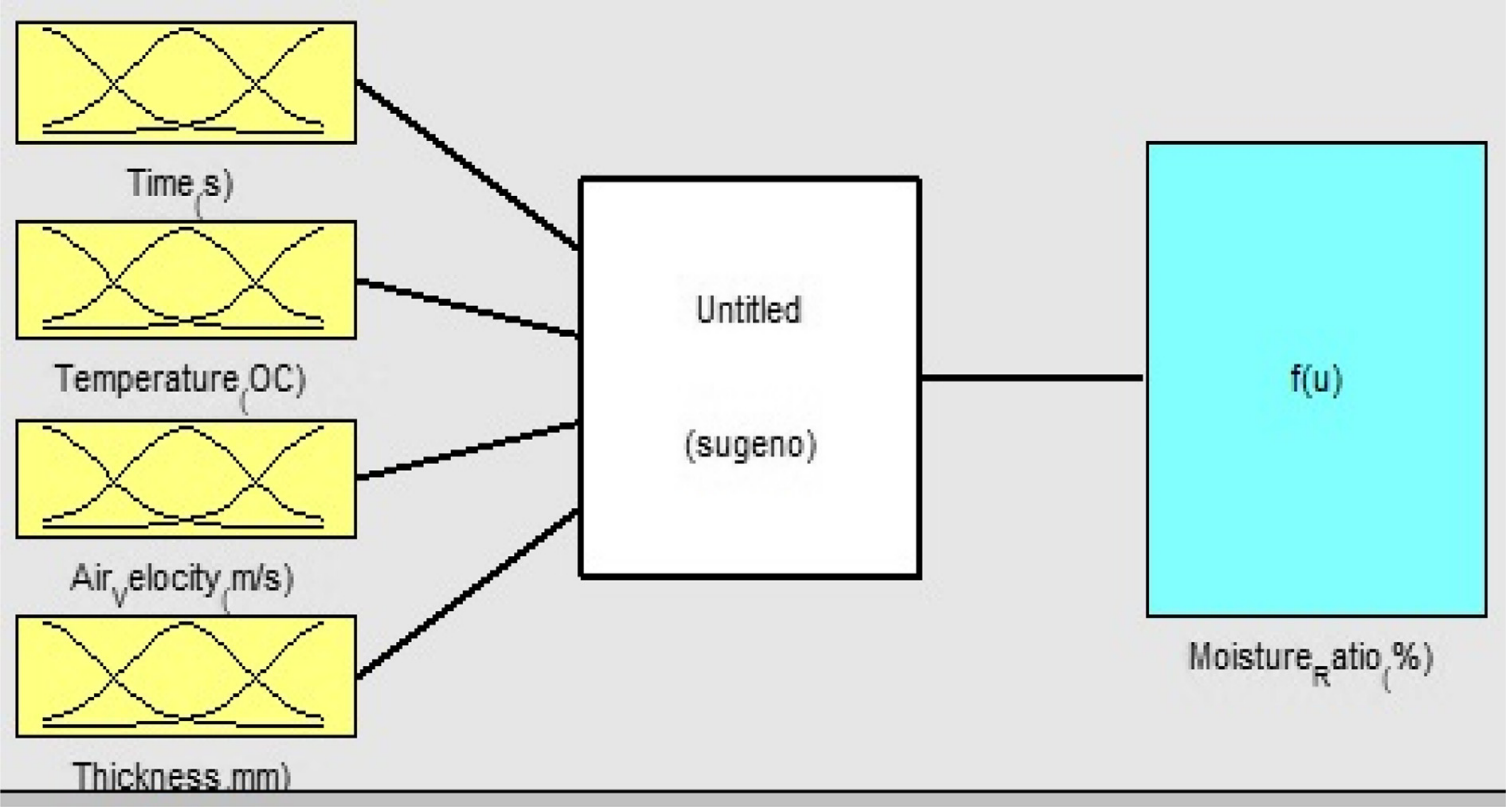
ANFIS in this study.

**Figure 4:**
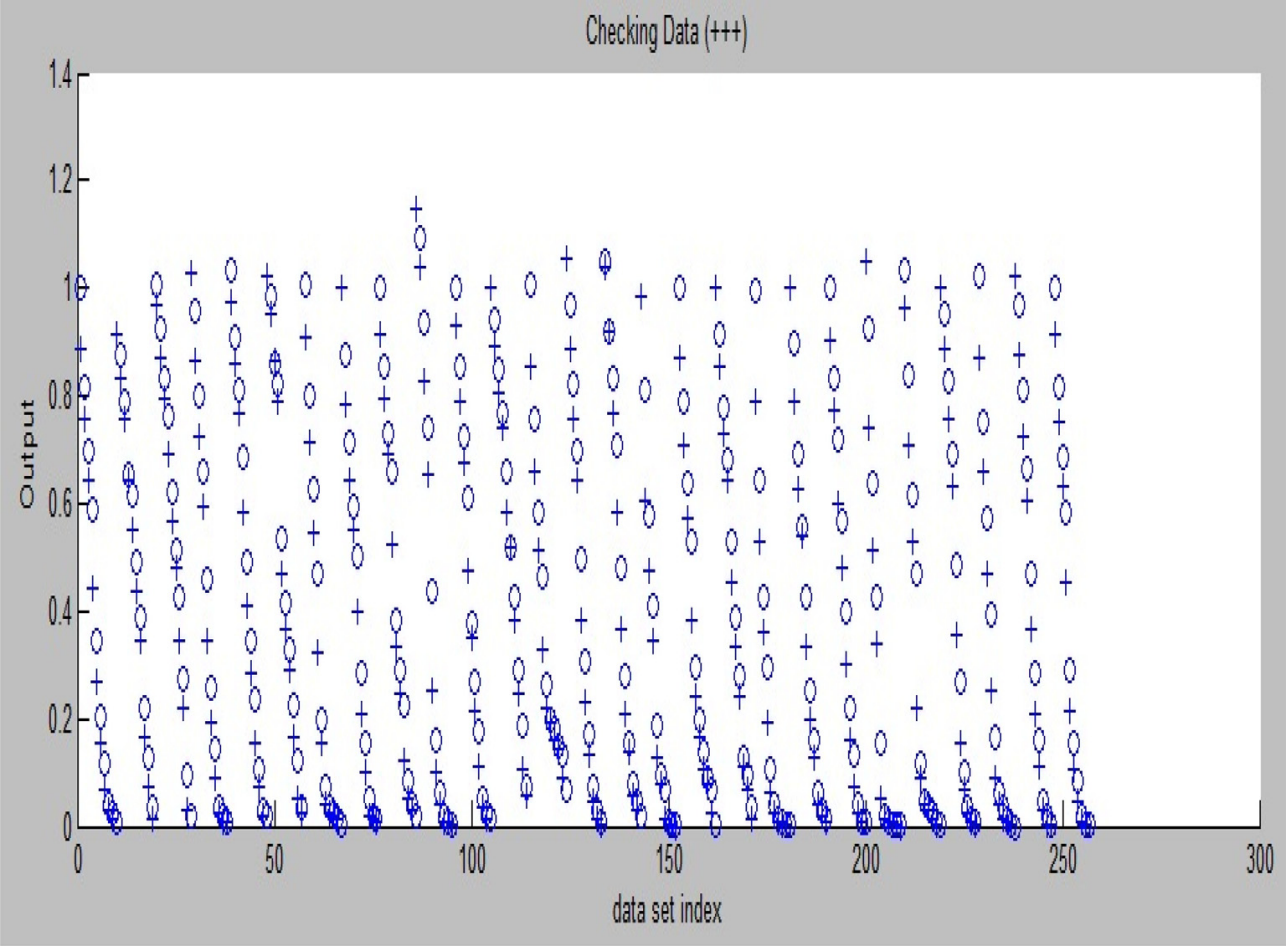
ANFIS partition.

The developed ANFIS layout from the experimental data is shown in Figure 3. There are 4 inputs (air temperature, drying time, air velocity, and yam slice thickness) and 1 output (moisture ratio). The maximum time for the experiment was 780 min. The partitioning of the experimental drying data into various data sets in this study was represented in Figure 4, where the training data (odd number of experimental drying data) set are represented with an 'o' sign and checking data (even number of experimental drying data) set are represented with a '+' sign. The data distribution as seen in Figure 4 showed that the spread of the data was interwoven. This spread was important so that ANFIS can easily understand the dynamics that exist in data during training and testing operations.

**Figure 5:**
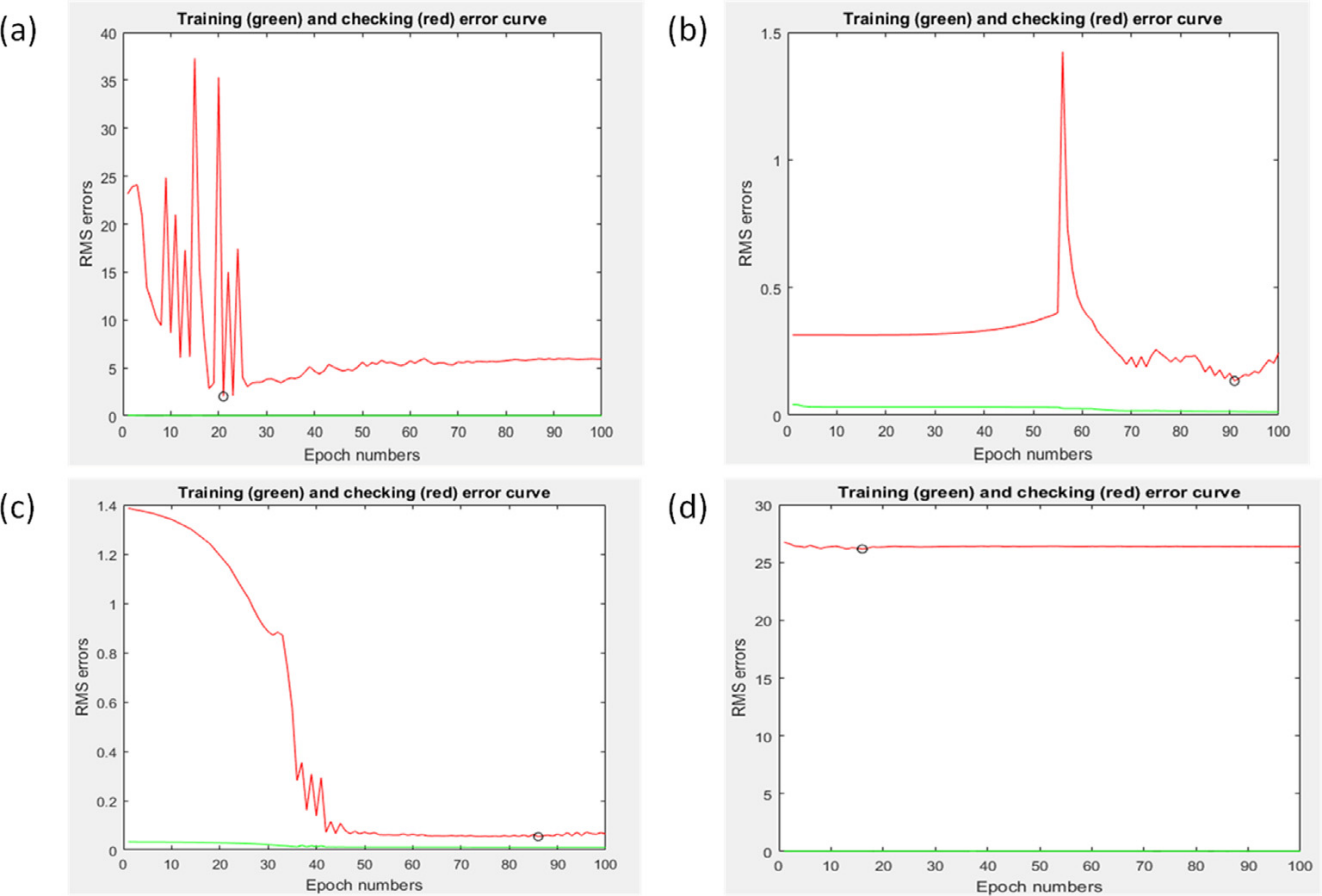
Effect of membership function type (a) Trapmf, (b) gaussmf, (c) dsigmf and (d) gbellmf on the speed of error convergence or simulation.

The modeling and prediction were mainly driven by ANFIS parameters, namely; the type of membership function, number of membership function, and minimum epoch number, amongst others. The optimum combination of these parameters will give an ANFIS structure or architecture that has acceptable effectiveness. In this study, the number of membership functions was fixed at 4, the epoch number was fixed at 100 while the effect of three types of membership function including trapmf, gbellmf, gaussmf, and dsigmf was tried. This was done to establish an ANFIS structure with optimum simulation speed in one part and ANFIS structure that will not overfit the training data set rather than learning from them. Details of the investigation of optimum ANFIS structure in this study were presented in Figure 5. It can be readily established from Figure 5 that the gbell membership function performed better in terms of speed of convergence when compared to the other tested membership function types. The ANFIS architecture of the gbell membership function has a structure that is optimum at eighteen (18) epochs or iteration while the trap, gauss and dsig membership function have their optimum structure at twenty-two (22), ninety-two (92), and eighty-seven (87) epochs, respectively. Therefore, the use of the gbell membership function is employed for the rest of the ANFIS modeling and prediction experiment.

**Figure 6:**
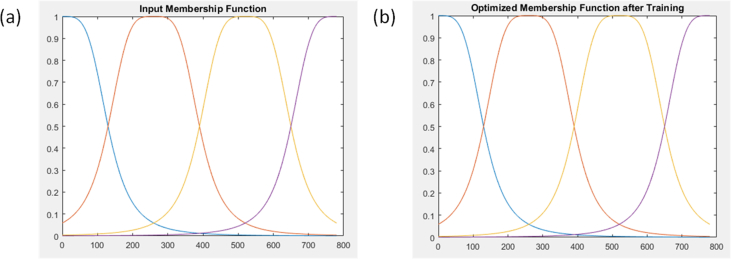
The shape of the (a) initial membership function and (b) refined membership function.

**Figure 7:**
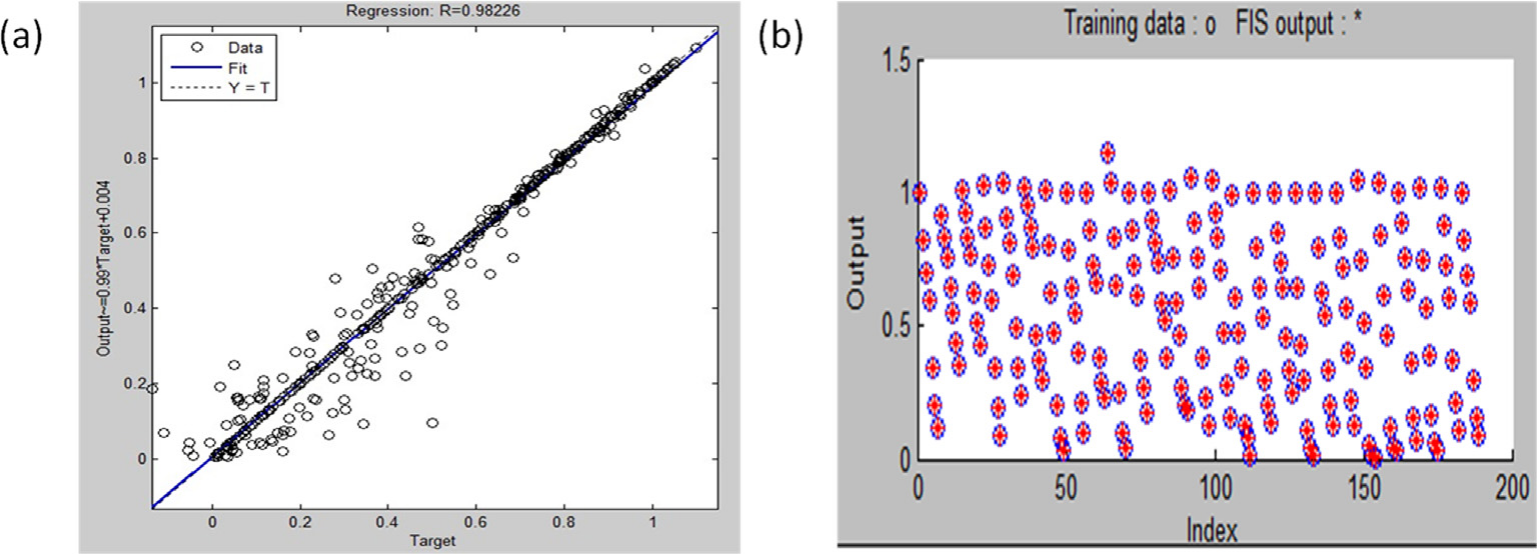
The (a) parity graph and (b) modeling efficiency of ANFIS.

Consequently, the initial shape of the initialized and optimum gbell membership function is represented in Figure 6 (a) and (b). In ANFIS and FIS, the shape of the membership function (universe of discourse) is important in giving a reliable data approximation (prediction). During developing a suitable ANFIS architecture, the membership function undergoes refinement from its original shape into an optimized shape because of the adaptive nature of ANFIS. This could be seen in Figure 6, where the crossing point in the shape of the refined membership function has slightly changed when compared with the shape of the original membership function. The original membership function's universe of discourse crossed at 0.5000 in the y-axis while that of refined membership function crossed at 0.4800 in the y-axis, hence a change in shape. Furthermore, the prediction efficiency of the optimum developed ANFIS structure is represented in Figure 7 (a) and (b). The efficiency was indicated with the correlation coefficient, which is the square root of the coefficient of determination value. Just like the coefficient of determination, the closer the value of the correlation coefficient to unity the better the data prediction ability. Figure 7 showed that the ANFIS structure had a correlation coefficient (R^2^) of 0.98226 and a calculated RMSE of 0.01702. A model with high$\;{\text{R}}^{2}$ and low RMSE signifies a good fit or performance (Khodabakhsh et al., 2015). Apart from the quality of the data under consideration, a typical ANFIS prediction efficiency also depends on the number of input data rows and columns. Although, large rows of data improved the accuracy of ANFIS. In the case of many input data columns, the application of exhaustive search is recommended for the establishment of only input data columns that are most influential on the output data. Figure 7 (b) showed the point to point prediction of experimental data by ANFIS optimum architecture, hence the modeling efficiency.

**Figure 8:**
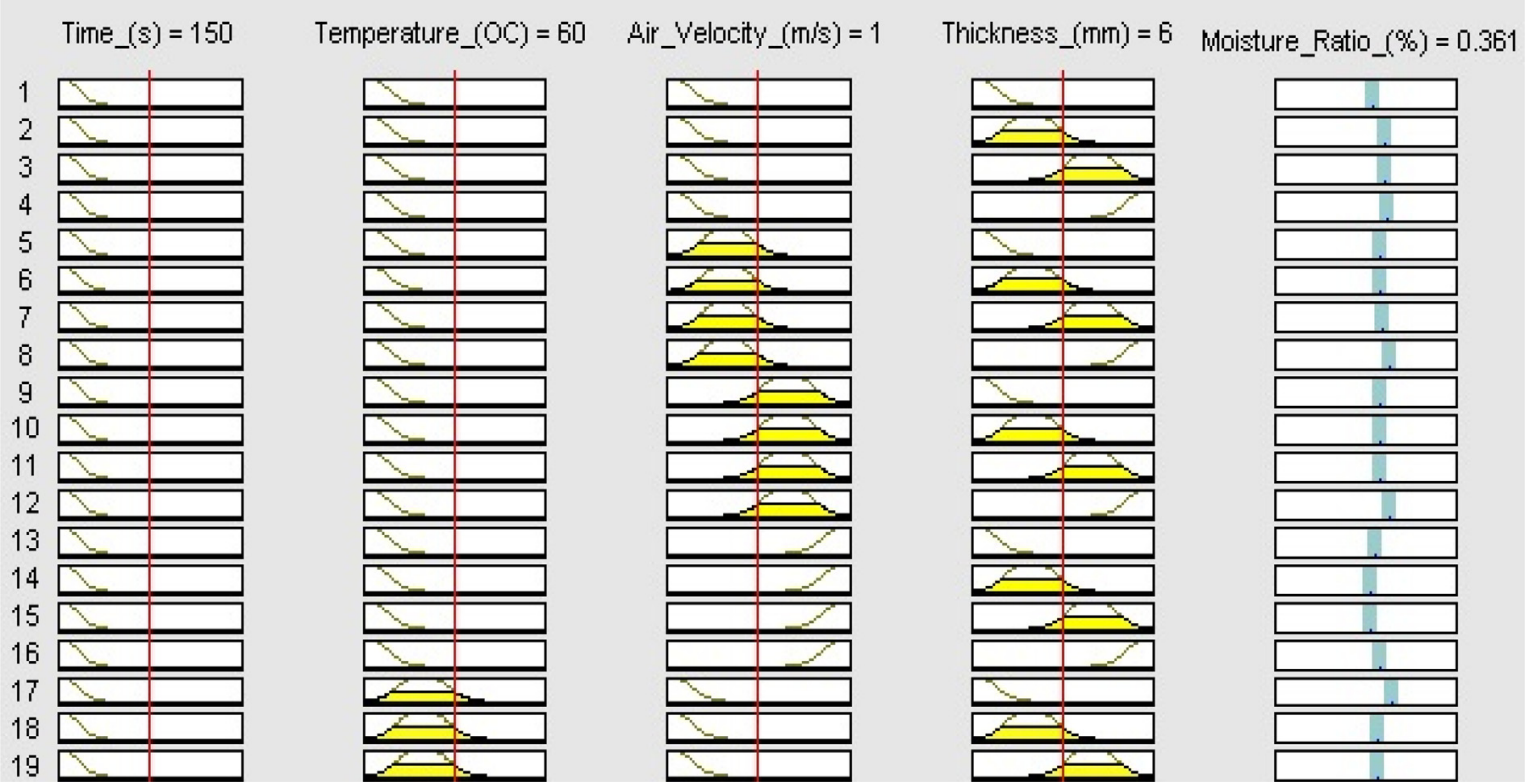
Rule viewer for the effect of the experimental condition on the moisture ratio.

**Figure 9:**
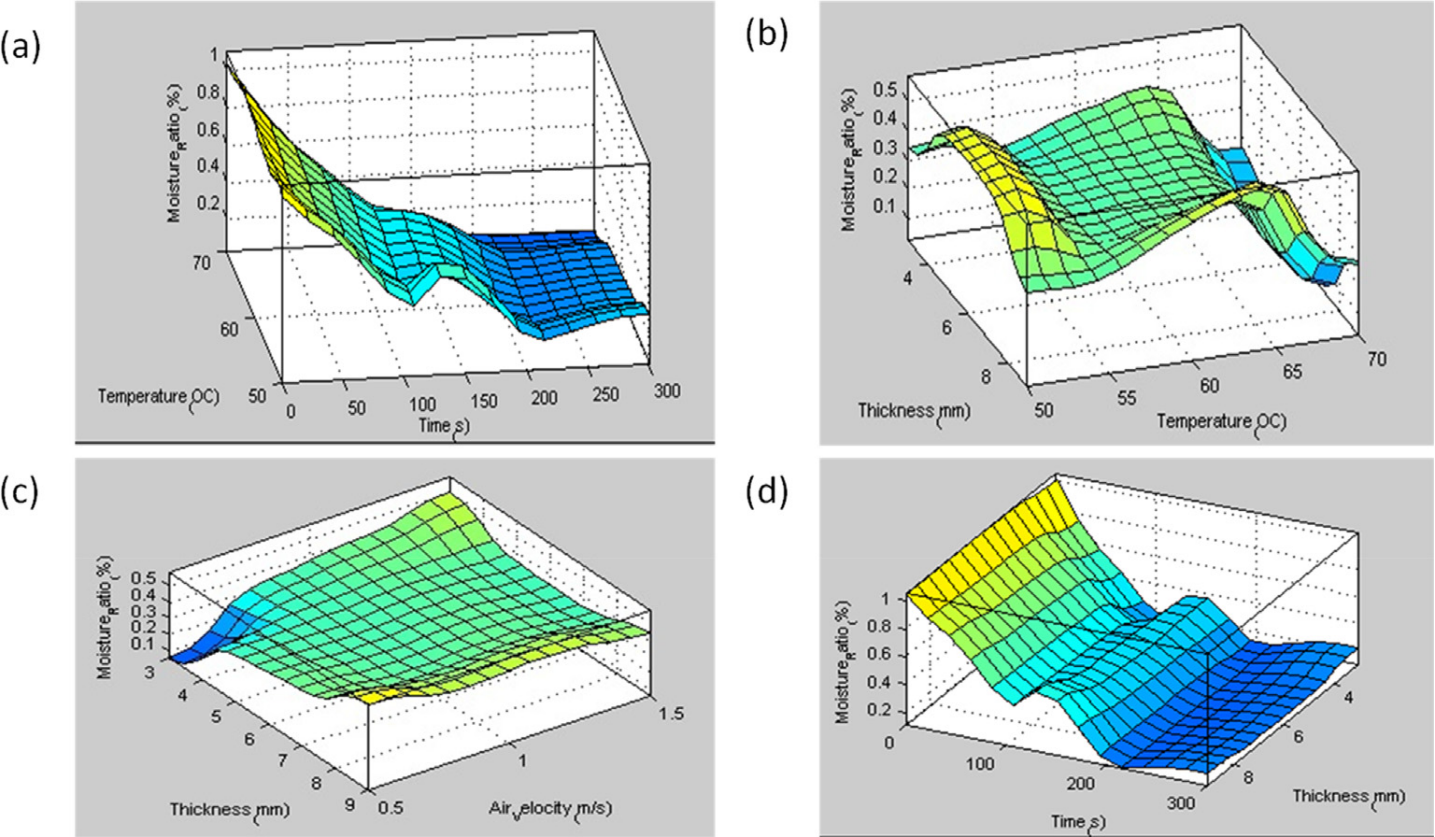
Graph of relationship between Moisture ratio versus input data's; (a) temperature and time, (b) thickness and temperature, (c) thickness and air velocity, (d) time and thickness.

Additionally, ANFIS automatically formulate rules for its data prediction activity. In this study, ANFIS formulated 250 rules to model and predict the experimental drying data. This large number of rules is consequent of the number of the experimental data points including the rows and the columns. Here, 513 experimental data point was used for ANFIS modeling. Figure 8 represents some of the ANFIS formulated rules. The relationship between ANFIS modeled inputs and output data in this study is represented in Figure 9(a-d). The figure showed the dependence of different input parameters against the moisture ratio, which is the sole output parameter. This result is in line with other reports. Kaveh et al. (2018b) reported a high coefficient of determination (R^2^ = 0.9974) for ANFIS was applied to the simulation of the moisture ratio of potato under a convective hot air dryer. Abbaspour-Gilandeh et al. (2019) also reported a coefficient of determination of 0.9997 when ANFIS was used for predicting the moisture ratio of quince fruit under a hot air dryer.

### 3.2 Effective diffusivity of yam slices

From Eq. (14), the natural logarithm of the moisture ratio (lnMR) was plotted against drying time (t), from which slope (K) was used to calculate the effective diffusivity (D_eff_) for all drying parameters considered (Table 3). Generally, the effective diffusivity increased with an increase in the air velocity (0.5–1.5 m/s), increased with an increase in the temperature (50–70 °C) and increased with an increase in the thickness of yam slice (3–9 mm). The lowest and highest effective diffusivity was found to be 6.382E -09 m^2^/s and 1.641E -07 m^2^/s respectively. Since effective diffusivity describes the rate at which moisture moves from the center of the slab geometry to the surface before being evaporated, making these observations expected. A similar increase was reported by Falade et al. (2007) for *Dioscorea rotundata* (0.829 ×10^−6^ to 1.121×10^−5^ m^2^/s) and *Dioscorea alata* (9.92×10^−8^ to 1.02×10^−7^ m^2^/s) with increase drying temperature and slice thickness, but air velocity has not yet been reported. Sridhar and Charles (2019), effective diffusivity for commercial grape (Kyoho) seeds was between 2.69×10^−8^ to 8.68×10^−8^ m^2^/s. Doymaz (2012) reported an increase in the effective diffusivity (1.31×10^−10^ to 3.66×10^−10^ m^2^/s) for sweet potato slice as infrared power levels were increased. Srikanth et al. (2019) also reported a similar increase in the effective diffusivity of elephant foot yam from 6.69×10^−8^ to 3.41×10^−7^ m^2^/s with increased drying temperature. The values reported for the effective diffusivity were in the same range as the values estimated.

**Table 3:** Effect of temperature, air velocity on effective diffusivity of yam slice at different thickness.

	3 mm		6 mm		9 mm	
	$D_{eff}$(m^2^/s)	R^2^	$D_{eff}$(m^2^/s)	R^2^	$D_{eff}\;$(m^2^/s)	R^2^
0.5 m/s						

50 °C	6.382E -09	0.979	1.823E -08	0.987	4.102E -08	0.991
60 °C	8.205E -09	0.956	2.188E -08	0.976	4.102E -08	0.904
70 °C	9.117E -09	0.932	2.918E -08	0.953	5.742E -08	0.951

1 m/s						

50 °C	6.382E -09	0.945	1.823E -08	0.989	3.281E -08	0.988
60 °C	4.558E -09	0.715	2.918E -08	0.986	4.922E -08	0.918
70 °C	1.003E -08	0.834	3.647E -08	0.964	5.742E -08	0.968

1.5 m/s						
50 °C	7.293E -09	0.932	2.553E -08	0.957	4.922E -08	0.967
60 °C	1.094E -08	0.970	2.188E -08	0.923	4.922E -08	0.885
70 °C	9.117E -09	0.965	4.741E -08	0.977	1.641E -07	0.699

### 3.3 Activation energy of yam slices

The activation energy is the required energy for moisture diffusion in products. A plot of the natural logarithm of the estimated effective diffusivity (lnD_eff_) versus the reciprocal of the absolute temperature (1/k) was used to calculate the activation energy (KJ/mol) as shown in Table 4 with its correlation values. Generally, the activation energy obtained using the Arrhenius equation increased with an increase in the air velocity, but within the different slice thickness considered the increase or decrease in the activation energy was fluctuating. The activation energy exists within the range of 10.59–54.93 KJ/mol. This is reported by some existing literature; Falade et al. (2007) for *Dioscorea alata* and *Dioscorea rotundata* (25.26–72.47 KJ/mol); Srikanth et al. (2019) for elephant foot yam (25.18–32.46 KJ/mol); Doymaz (2011) for fresh potato slice and blanched potato (22.7 and 23.2 KJ/mol). The dissimilarities in the values may be due to some factors as reported by Srikanth et al. (2019); ripening stage, operating conditions, tissue structure and components, variety, and size.

**Table 4:** Effect of air velocity on the activation energy ($E_{a}$) of yam slice at different thickness.

	$E_{a\;}$(kJ/mol)	R^2^
0.5 m/s		

3 mm	16.53	0.954
6 mm	21.62	0.978
9 mm	15.34	0.734

1 m/s		

3 mm	20.33	0.310
6 mm	32.07	0.965
9 mm	25.91	0.945

1.5 m/s		

3 mm	10.59	0.310
6 mm	10.59	0.310
9 mm	54.93	0.734

### 3.4 Rehydration ratio

**Figure 10:**
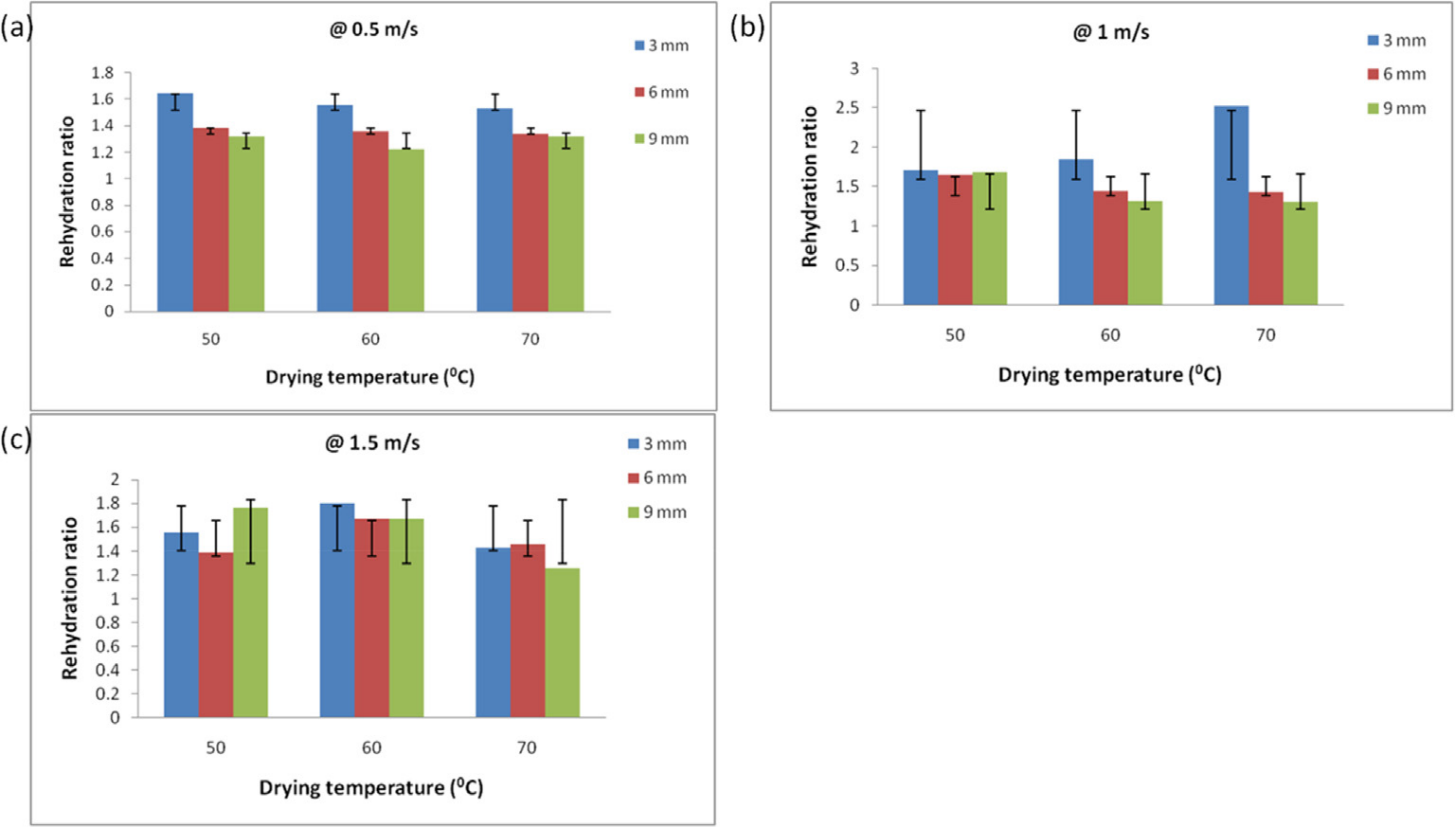
Rehydration curves for yam slices dried at air temperatures (50, 60, and 70 °C), slice thickness (3, 6, and 9 mm), and air velocity levels; (a) 0.5 m/s, (b) 1 m/s, and (c) 1.5 m/s.

Rehydration ratio is a quality parameter that establishes the ability of food material to return to its original shape and it also shows the degree of the cell destruction during drying as affected by its operating conditions (Doymaz, 2011; Srikanth et al., 2019). Rehydration ratio values for yam slices were estimated using Eq. (17), as shown in Figure 10 (a-c). It shows that the drying conditions used (air velocity, temperature, and thickness) had a significant effect on the rehydration ratio. Air velocity of 0.5 m/s, the temperature of 70 °C, and a thickness of 3 mm had the lowest rehydration ratio value (Figure 10a). Whereas, air velocity (1.5 m/s), temperature (70 °C), and slice thickness (3 mm) showed the highest rehydration ratio (Figure 10c). Slice thickness of 9 mm was only noticed to have a steady decrease in rehydration ratio as temperature increased (50–70 °C) at all air velocity levels, while 3 mm slice thickness decreased at 1 m/s (Figure 10b), and increased at 1.5 m/s air velocity. Doymaz (2012) reported an increase in rehydration ratio as infrared power levels (product temperature) was increased but decreased with an increase in power levels from 146 to 167 W for potato slice. Srikanth et al. (2019) reported that rehydration ratio increased with an increase in the drying temperature but at 40 °C lower rehydration values were observed for elephant foot yam. These fluctuations might be due to part of the yam cut and temperature used during drying.

### 3.5 Drying characteristics

**Figure 11:**
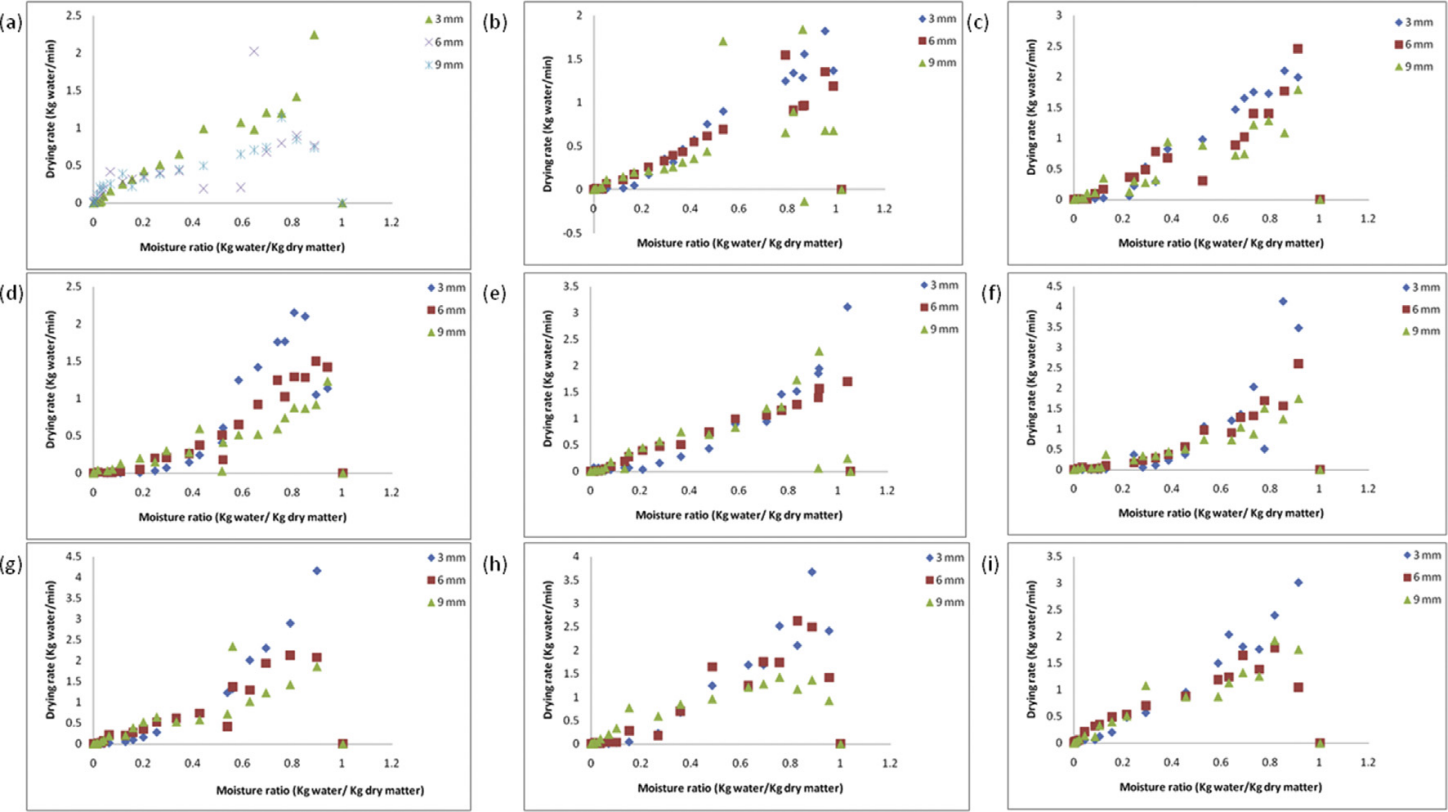
Drying rates versus moisture ratios at temperature and air velocity levels (a: 50 °C and 0.5 m/s, b: 50 °C and 1 m/s, c: 50 °C and 1.5 m/s, d: 60 °C and 0.5 m/s, e: 60 °C and 1 m/s, f: 60 °C and 1.5 m/s, g: 70 °C and 0.5 m/s, h: 70 °C and 1 m/s, i: 70 °C and 1.5 m/s) for 3 mm, 6 mm, and 9 mm yam slice thickness.

**Figure 12:**
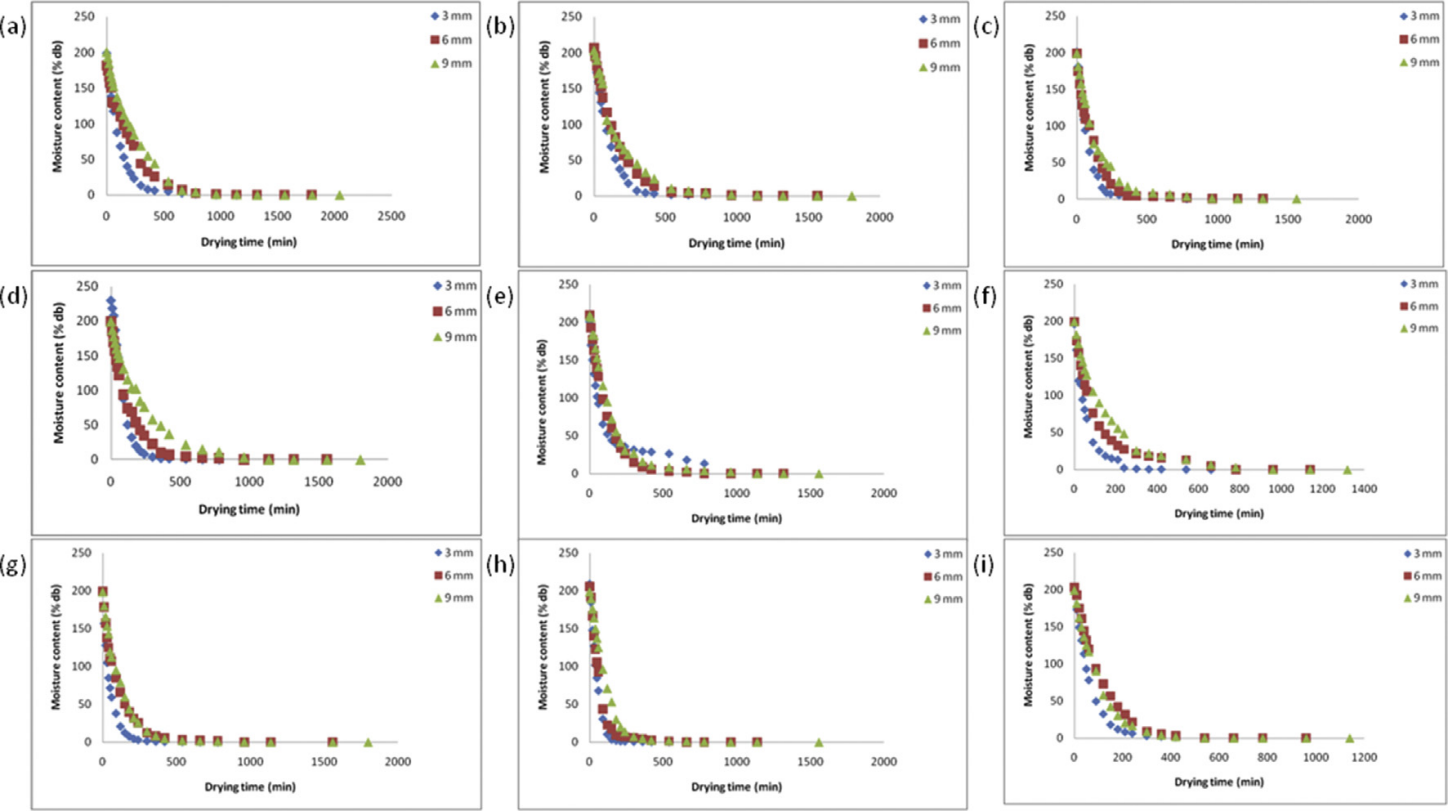
Moisture content (% dry basis) versus drying time at temperature and air velocity levels (a: 50 °C and 0.5 m/s, b: 50 °C and 1 m/s, c: 50 °C and 1.5 m/s, d: 60 °C and 0.5 m/s, e: 60 °C and 1 m/s, f: 60 °C and 1.5 m/s, g: 70 °C and 0.5 m/s, h: 70 °C and 1 m/s, i: 70 °C and 1.5 m/s) for 3 mm, 6 mm, and 9 mm yam thickness slice.

Figures 11 and 12 show the relationship between the drying rate versus drying time and changes in moisture content with drying time at various parameters; air temperatures (50, 60, 70^o^c), air velocity (0.5, 1, 1.5 m/s), and thickness (3, 6, 9 mm) for yam slices. The drying rate and the moisture content decrease continually with drying time at all parameters considered. The results showed that the drying of yam slices exists solely in the falling rate period, which showed that the internal moisture diffusion phenomenon is dominant i.e. it controlled the drying process. The results were consistent with some related literature on drying of tuber crops; Falade et al. (2007) for yam, Doymaz (2011) for sweet potato, Ju et al. (2016) for yam. From the results in Figure 12, the air temperatures, air velocity, and thickness all had a significant effect on the moisture content of the yam slice as envisaged. The required drying time of samples were 780, 960, 1140, 1320, 1560, and 1800 min at air temperature, air velocity, and thickness combination of 70 °C × 1.5 m/s×3 mm down to 50 °C × 0.5 m/s× 9 mm. In drying, the air velocity influences the moisture removal from the surface of the product, it was recorded that air velocity of 1.5 m/s for all temperatures and thickness considered, gave a higher drying rate. The drying rates for 3 mm sample thickness was higher compared with 6 mm and 9 mm for all air temperature and velocities considered. The higher drying rate of yam slice for 3 mm might be due to the nearness of the center core of the slice to the surface making moisture diffusion faster. The higher drying rate with a decrease in slice thickness has been reported by Falade et al. (2007) and Doymaz (2012) for root and tubers.

## 4 Conclusion

The experiments were performed for different drying parameters; drying temperatures (50, 60, and 70^0^c), air velocities (0.5, 1, and 1.5 m/s), and slice thickness (3, 6, and 9 mm). Having analyzed the drying data with ANFIS, the following conclusions were established; ANFIS showed capability in modeling and prediction of drying data (R^2^ = 0.98226). The method was particularly useful because it was devoid of mathematical relationships and such methods are easily applicable in the industry through the creation of a lookup table. The gbell membership function performed better in terms of speed of convergence when compared with other tested membership functions in this study. The effective diffusivity increased with an increase in air velocity, air temperature, and thickness, and the values ranged between 6.382E -09 to 1.641E -07 m^2^/s. The activation energy increased with an increase in air velocity, but fluctuate within the air temperatures and thickness, and the values ranged between 10.59 to 54.93 KJ/mol. Air velocity of 1.5 m/s, temperature (70 °C) and thickness (3 mm) had the highest rehydration ratio, while the air velocity of 0.5 m/s, temperature (70 °C) and thickness (3 mm) had the lowest rehydration ratio. The drying rate increased with an increase in air temperature, air velocity, and decreased slice thickness. Drying proceeded entirely in the falling rate period.

## Declarations

### Author contribution statement

John O. Ojediran: Conceived and designed the experiments; Analyzed and interpreted the data; Wrote the paper.

Clinton E. Okonkwo: Conceived and designed the experiments; Performed the experiments; Analyzed and interpreted the data; Wrote the paper.

Abiola J. Adeyi, Oladayo Adeyi: Analyzed and interpreted the data; Wrote the paper.

Abiola F. Olaniran: Performed the experiments; Contributed reagents, materials, analysis tools or data.

Nana E. George: Performed the experiments.

Adeniyi T. Olayanju: Contributed reagents, materials, analysis tools or data.

### Funding statement

This research did not receive any specific grant from funding agencies in the public, commercial, or not-for-profit sectors.

### Competing interest statement

The authors declare no conflict of interest.

### Additional information

No additional information is available for this paper.
